# A Rare Case of Malignant Insulinoma Associated With Gastrointestinal Bleed

**DOI:** 10.7759/cureus.64894

**Published:** 2024-07-19

**Authors:** William K Boateng, Kosisochukwu J Ezeh, Youssef Botros, Etan Spira, Tingliang Shen

**Affiliations:** 1 Internal Medicine, Jersey City Medical Center, Jersey City, USA; 2 Gastroenterology and Hepatology, Jersey City Medical Center, Jersey City, USA; 3 Pathology, Jersey City Medical Center, Jersey City, USA

**Keywords:** esophagogastroduodenoscopy (egd), neuroendocrine tumors, gastrointestinal bleed, malignant insulinoma, insulinoma

## Abstract

A gastrointestinal bleed (GIB) in the setting of metastatic insulinoma is a rare phenomenon. It appears that cases of metastatic insulinoma causing GIB are rare, often influenced by the tumor's location. Our case involves an 82-year-old male with dementia and a history of recurrent hypoglycemia, presenting with an episode of altered mental status. The patient exhibited hypoglycemia alongside a melena episode and anemia. Diagnostic criteria, including Whipple’s triad, confirmed endogenous insulin production. Computed tomography (CT) showed a left paraaortic/retroperitoneal mass. Esophagogastroduodenoscopy (EGD) visualized an extrinsic mass at the gastric body, which caused an ulcerated surface that was treated with clipping and hemostasis. The patient’s recurrent hypoglycemic episodes were treated with glucose, while his GIB was managed with hemostasis and clipping. However, the patient was not a surgical candidate, and further medical treatment was ceased by the family.

## Introduction

Insulinoma is a common neuroendocrine tumor that can secrete endogenous insulin, resulting in episodes of recurrent hypoglycemia. Insulinomas are commonly found in the pancreas [1]. Malignant insulinoma occurs in 5.8% of cases [2]. Functioning extrapancreatic insulinomas are rare (incidence <2%) and are commonly found in the duodenal wall [3]. Patients generally present with the classic symptoms of low plasma glucose, symptoms of hypoglycemia, and hypoglycemia that resolve after glucose administration. Gastrointestinal bleed (GIB) in association with malignant insulinoma is extremely rare, with only one other case report found in the literature. Management involves controlling the GIB, managing the hypoglycemia, and ultimately treating the insulinoma with medical or surgical intervention.

Herein, we describe a case of a patient with insulinoma that was associated with GIB.

## Case presentation

We present an 82-year-old male with a history of dementia, hypertension, and chronic kidney disease who came in after an episode of altered mental status.

Emergency medical service (EMS) reports the patient’s family noticed that the patient was conversing, suddenly became altered mid-conversation, and stopped responding. The patient was brought in by EMS, and the blood glucose (BG) level was 23 mg/dL in the Emergency Department. Repeat BG after administration of intravenous (IV) 10% dextrose solution (D10) was 75 mg/dL. The physical exam revealed the patient was alert and oriented only to the person and not time or place. The rest of the exam was otherwise unremarkable.

On the day of admission, the patient was found to have orthostatic hypotension (BP 100/60 mmHg) after an episode of a witnessed fall. The patient required a fluid bolus. The patient then had an episode of a large melenic stool after his first bowel movement. The hemoglobin (Hgb) on admission was 13 g/dL but dropped to 9.8 g/dL after the melenic episode. The patient received one unit of packed red blood cells (pRBCs). The patient’s BG was labile and ranged from 35 to 171 mg/dL. The patient was started on continuous IV D10 with normal saline at a rate of 75 cc/hour. He was given another fluid bolus and a proton pump inhibitor drip. The patient was transferred to the medical intensive care unit (MICU) for close monitoring. 

The patient’s serum glucose was 46 (normal 74-106) mg/dL at the time lab results were gathered. Insulin antibody was less than 0.4 (normal range less than 0.4) U/mL, serum insulin of 27 (normal range ≤20) mIU/mL, serum proinsulin of 686.5 (normal range ≤18.8) pmol/L, and C-peptide of 5.68 (normal range 0.80-3.85) ng/mL. Sulfonylurea drug levels were negative. Gastrin was not measured due to the patient being treated with proton pump inhibitors.

The patient required a total of two additional units of blood due to the Hgb being below 7 g/dL on two separate occasions. There were no episodes of melena, hematochezia, or hematemesis, and the patient was hemodynamically stable. After three days in the MICU, the patient was sent to the routine medical floor for further management. The patient’s BG continued to be labile, but an increase in the patient’s D10 to a rate of 125 cc/hour resulted in fewer episodes of hypoglycemia. 

**Figure 1:**
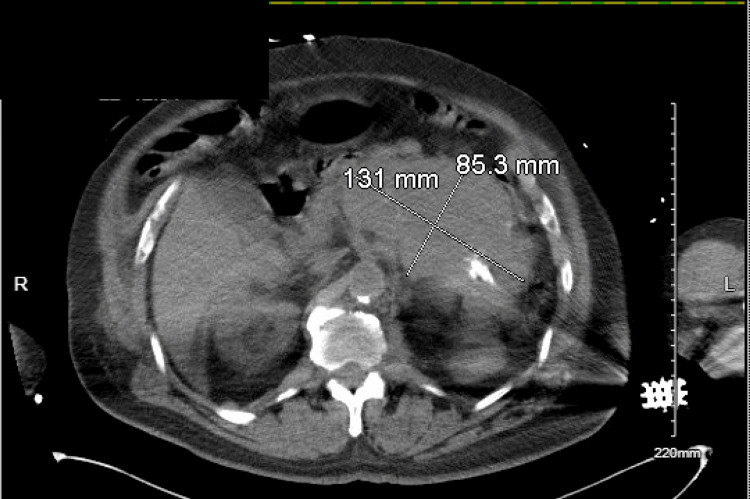
CT finding of a left paraaortic/retroperitoneal mass encasing the left side of the aorta, causing hydronephrosis of the left kidney CT: Computerized tomography

Computerized tomography (CT) of the abdomen and pelvis without contrast showed a left paraaortic/retroperitoneal mass encasing the left side of the aorta, causing hydronephrosis of the left kidney. The size of the mass was at least 8 x 13 x 17 cm, and calcifications were seen within it (Figure 1). Esophagogastroduodenoscopy (EGD) showed a normal esophagus, a small hiatal hernia, clotted blood in the fundus without an active bleed, and granular gastric mucosa in the antrum was biopsied. The biopsy of the antrum was positive for *Helicobacter pylori* (*H. pylori*). 

**Figure 2:**
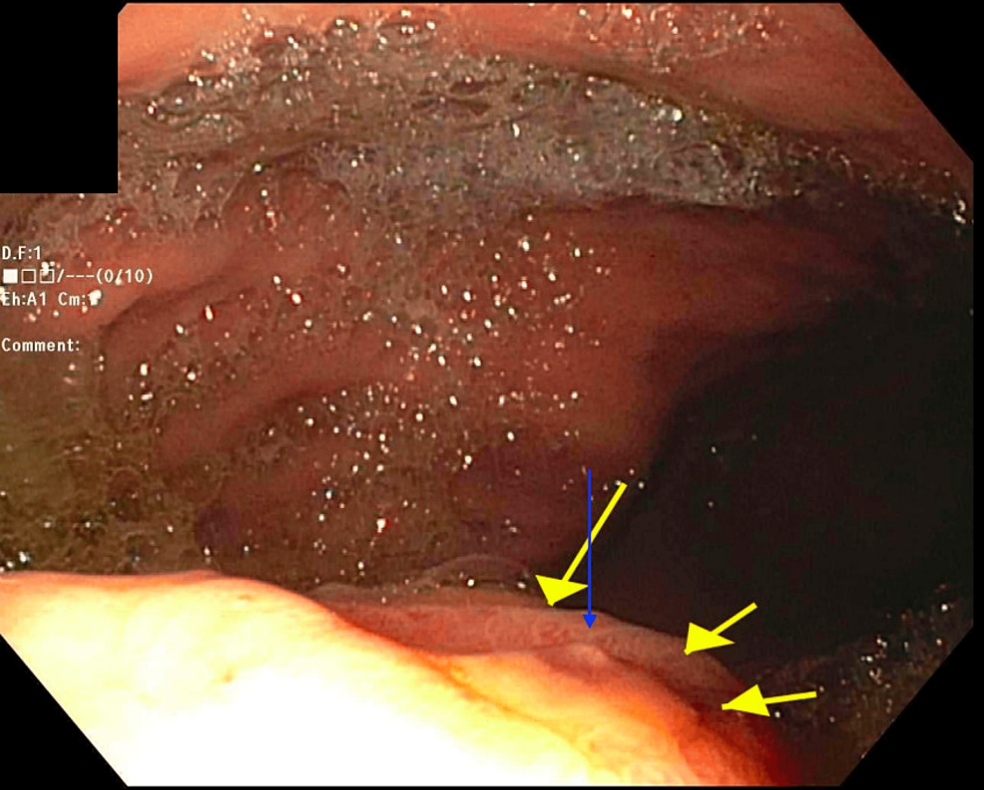
EGD finding of an ulcerated area of the extrinsic compression at the gastric body (yellow arrows); non-bleeding visible vessel (Forrest Class IIa) (blue arrow) EGD: Esophagogastroduodenoscopy

**Figure 3:**
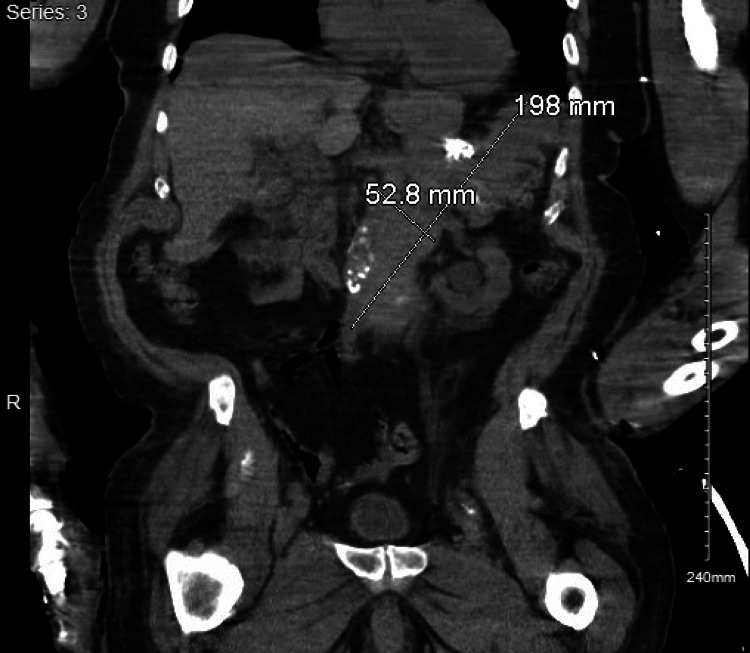
CT finding of a retroperitoneal and left upper quadrant mass with lymphadenopathy CT: Computerized tomography

**Figure 4:**
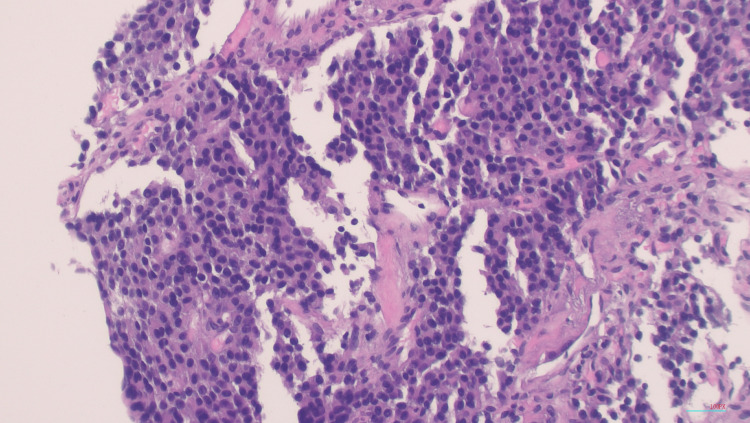
H&E stain (100x magnification) The tumor is composed of uniform small ovoid to cuboidal nuclei with fine chromatin and eosinophilic finely-granular cytoplasm arranged in organoid or solid pattern. Mitotic figures are absent. H&E: Hematoxylin and eosin

**Figure 5:**
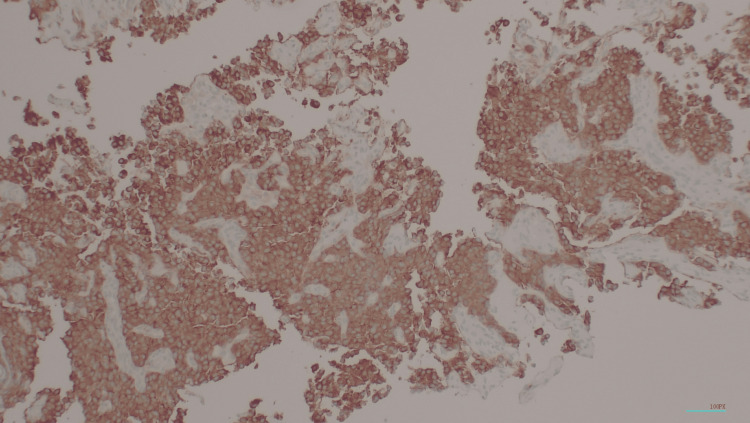
Immunohistochemical stain of synaptophysin (50x magnification)

**Figure 6:**
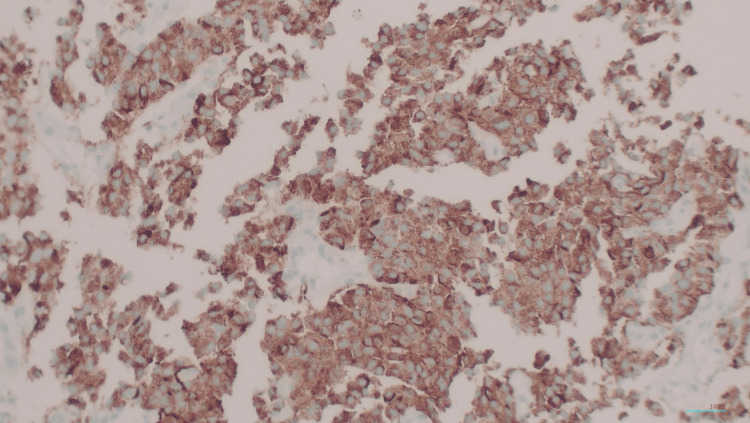
Immunohistochemical stain of chromogranin (100x magnification) The tumor cells show diffuse intense cytoplasmic reactions.

A second-look EGD showed an extrinsic compression in the gastric body. The extrinsic mass was characterized by an ulcerated surface. At the site of the mass, there was a non-bleeding gastric ulcer with a non-bleeding vessel that was clipped and treated with hemostasis (Figure 2). The antrum was noted to be erythematous. Interventional radiology was consulted for a biopsy of the mass. A repeat CT of the abdomen and pelvis reiterated a retroperitoneal mass along with lymphadenopathy that was concerning for metastatic disease (Figure 3). A biopsy of the retroperitoneal mass revealed a well-differentiated neuroendocrine neoplasm (Figure 4). Tumor cells were strongly positive for synaptophysin (Figure 5) and chromogranin (Figure 6). Tumor cells were negative for CD138 (excluding plasmacytoma) and negative for signal transducer and activator of transcription 6 (STAT6) (excluding solitary fibrous tumor). Ki67 for grading tumor was 2%. Due to the patient’s clinical findings of hypoglycemia, evidence of overproduction of endogenous insulin, and confirmation of tissue biopsy, the diagnosis of insulinoma was concluded.

Surgery was consulted, but they deemed that no acute surgical intervention was indicated because the patient was not a surgical candidate. Oncology discussed the possibility of further evaluation and treatment. The patient’s family declined treatment and requested the patient be placed in hospice. The patient was ultimately discharged to a hospice facility. 

## Discussion

The incidence of extrapancreatic insulinomas is less than 2% [1]. We searched for publications in PubMed from the last 30 years and found only one other reported case of an upper gastrointestinal bleed (UGIB) in patients with malignant insulinoma [4]. In the case of Medina-Franco et al., EGD was attempted with clipping and argon beam coagulation but was unsuccessful. The case was then managed surgically to maintain hemostasis. Surgery is generally the best treatment for insulinoma, based on tumor size, pathological features, and location [5].

Our case differs in that hemostasis was achieved endoscopically. Our patient’s extrapancreatic mass was located at the gastric body and resulted in a non-bleeding ulcerated lesion (Forrest Class IIa) with a visible vessel. The ulcerated lesion was treated with clipping and injection of diluted epinephrine. The patient required only one unit of pRBC the day after the EGD until discharge, compared to the three units in four days before the procedure. Our patient was not a surgical candidate, and medical treatment was denied by the family. 

Extrapancreatic insulinomas are generally found in the duodenal wall but can also be found in the spleen, stomach, duodenohepatic ligament, or jejunum [3,6]. Despite the few reported cases of ectopic insulinoma, there are rarely any that are associated with GIB based on literature review.

The chronic gastritis, possibly caused by the positive *H. pylori*, may have played a role in the susceptibility of our patient's bleed. However, the presence of the ulcer and vessel located directly at the site of the extrinsic mass in the body cannot be overlooked. Gastrinoma is another neuroendocrine tumor that can cause ulcerations and bleeding. Gastrin was not measured in our patient due to the high-dose proton pump inhibitors already administered on presentation, which could have altered results [7]. Gastrinoma and insulinoma presenting concurrently are rare. In our literature review, they are generally found in cases with the syndrome, multiple endocrine neoplasia type 1 (MEN1). In patients with MEN1, each hormone (insulin and gastrin) is secreted by different tumors [8,9].

The presentations of GIB in both cases were likely due to the location of the mass being near the stomach. More research concerning similar extrapancreatic insulinomas would aid in finding an association between the location, resulting GIB, and the appropriate management.

## Conclusions

In conclusion, the management of both insulinoma and GIB remains to treat hypoglycemia and control the source of the bleeding, respectively. Typically, extrapancreatic insulinomas are located within the duodenal wall. However, in both the review and our case, the mass was located proximal to the stomach, contributing to GIB. This suggests a potential correlation between the anatomical location of extrapancreatic insulinomas and the presentation of UGIB. Although surgical resection remains the definitive treatment for achieving hemostasis and addressing insulinoma symptoms, our findings demonstrate that endoscopic techniques can be a viable alternative in patients who are not suitable candidates for surgery. More research is required to understand the relationship between the site of extrapancreatic insulinomas and the risk of UGIB, as well as to optimize management strategies for these cases.
